# Neurobiological Correlates of EMDR Monitoring – An EEG Study

**DOI:** 10.1371/journal.pone.0045753

**Published:** 2012-09-26

**Authors:** Marco Pagani, Giorgio Di Lorenzo, Anna Rita Verardo, Giampaolo Nicolais, Leonardo Monaco, Giada Lauretti, Rita Russo, Cinzia Niolu, Massimo Ammaniti, Isabel Fernandez, Alberto Siracusano

**Affiliations:** 1 Institute of Cognitive Sciences and Technologies, Consiglio Nazionale delle Ricerche (CNR), Rome, Italy; 2 Department of Systems Medicine, University of Rome “Tor Vergata”, Rome, Italy; 3 EMDR Italy Association, Bovisio Masciago (MI), Italy; 4 Department of Developmental and Social Psychology, “Sapienza University of Rome”, Rome, Italy; 5 International Psychoanalytical Association, “Sapienza University of Rome”, Rome, Italy; Max Planck Institute of Psychiatry, Germany

## Abstract

**Background:**

Eye Movement Desensitization and Reprocessing (EMDR) is a recognized first-line treatment for psychological trauma. However its neurobiological bases have yet to be fully disclosed.

**Methods:**

Electroencephalography (EEG) was used to fully monitor neuronal activation throughout EMDR sessions including the autobiographical script. Ten patients with major psychological trauma were investigated during their first EMDR session (T0) and during the last one performed after processing the index trauma (T1). Neuropsychological tests were administered at the same time. Comparisons were performed between EEGs of patients at T0 and T1 and between EEGs of patients and 10 controls who underwent the same EMDR procedure at T0. Connectivity analyses were carried out by lagged phase synchronization.

**Results:**

During bilateral ocular stimulation (BS) of EMDR sessions EEG showed a significantly higher activity on the orbito-frontal, prefrontal and anterior cingulate cortex in patients at T0 shifting towards left temporo-occipital regions at T1. A similar trend was found for autobiographical script with a higher firing in fronto-temporal limbic regions at T0 moving to right temporo-occipital cortex at T1. The comparisons between patients and controls confirmed the maximal activation in the limbic cortex of patients occurring before trauma processing. Connectivity analysis showed decreased pair-wise interactions between prefrontal and cingulate cortex during BS in patients as compared to controls and between fusiform gyrus and visual cortex during script listening in patients at T1 as compared to T0. These changes correlated significantly with those occurring in neuropsychological tests.

**Conclusions:**

The ground-breaking methodology enabled our study to image for the first time the specific activations associated with the therapeutic actions typical of EMDR protocol. The findings suggest that traumatic events are processed at cognitive level following successful EMDR therapy, thus supporting the evidence of distinct neurobiological patterns of brain activations during BS associated with a significant relief from negative emotional experiences.

## Introduction

Post-traumatic conditions lead to derangement of memory and mood regulation possibly ending with a fear-driven response elicited by internal or external cues associated with a traumatic situation [1].

Investigations by positron emission tomography (PET) and single photon emission computed tomography (SPECT) have identified an impairment of the medial prefrontal cortex (mPFC), associated with a hyper-reactivity of the amygdalae, to constitute the core neural correlate of post-traumatic stress disorder (PTSD) [2]. On the other hand, several studies have provided evidence for the clinical efficacy of Eye Movement Desensitization and Reprocessing therapy (EMDR) in the treatment of PTSD [3]. EMDR is an information processing therapy for anxiety disorders that focuses on trauma elaboration or highly stressful recollections [4]. A distinct characteristic of EMDR is the use of alternating bilateral stimulation such as eye movement, tactile or auditory. The patient is asked to focus upon the traumatic memory image while simultaneously attending to an alternate stimulus for brief eye movements (right-left) sets of approximately 30 seconds. As a result EMDR has been included in many international trauma treatment guidelines [5]–[8] and in 2011 has also been shortlisted as evidence-based practice for the treatment of PTSD [9], anxiety and depression symptoms [10].

Recent studies have probed into EMDR's mechanism of action and its physiological and neurobiological substrate [11]–[15] providing some preliminary evidence of an association between functional changes and treatment efficacy. However, none of these studies succeeded in investigating real-time firing of brain neurons in response to the external stimuli induced by EMDR since the effects of therapy on brain activation/deactivation were only recorded before and after EMDR treatment. This has restricted the reported information to static conditions not describing in detail the dynamics of regional neuronal synchronization during EMDR sessions.

Electroencephalography (EEG) helps to overcome such limiting factors as it records brain electrical activity with a time resolution of milliseconds and with an acceptable capability to identify the sources of activity in the brain 3D space, especially by means of a medium to high-density array of electrodes [16].

The aim of the study was (i) to explore the technical feasibility of the on-line recording of whole EMDR sessions by means of EEG; (ii) to identify the regions activated either by the autobiographic recollection of the traumatic event (script) or during the bilateral ocular stimulation at EMDR sessions; (iii) to investigate possible changes in functional connectivity both as a result of EMDR therapy or comparing patients and healthy controls; (iv) to correlate such changes to neuropsychological scores.

Due to the exploratory nature of the present study, we analyzed and reported separately all activities for the different frequencies of the cerebral electric spectrum.

## Materials and Methods

### Subjects

Ten psychologically traumatised symptomatic patients were included in the study (mean age 33±10; 4 males, 6 females). Patients were referred to clinicians specialized in EMDR treatment (AV, GL, RR, IF) on the basis of the presence of major psychological trauma. Although all patients were clinically diagnosed as suffering from PTSD, due to logistics and patients’ refusal to long and elaborate procedures, no categorical diagnosis could be made according to DSM-IV-TR criteria. Traumas consisted in sexual abuse (5), grief and loss trauma (3), abortion related trauma (1) and severe physical abuse (1) and EMDR sessions focused on these specific life events. Ten healthy subjects comparable for age and gender (mean age 37±7; 5 males, 5 females) and aware of the study agreed to participate and to act as controls of their own free will. In all of them the index trauma chosen was the one with the highest impact on their memories. The major distinction between patients and controls was the lack of trauma-related symptoms in the latter group. Exclusion criteria included a track record of clinically diagnosed psychiatric disorders and a score of the psychological response to the stressful index trauma (Impact of Event Scale total score, intrusion + avoidance) <26, i.e. less than a moderate psychological response to trauma. Prior to entering the study, all participants were informed of the procedures and asked to subscribe to the Declaration of Helsinki.

### Study Design

The study was entirely carried out in the therapy room of a private clinic to which all patients were referred for treatment. The room was quiet, light and airy and clinicians and patients, as well as controls, were comfortable enough to establish a therapeutic alliance. During the first session the clinician confirmed the presence of a major psychological trauma and the persistence of the related symptoms over time. All subjects were asked to record as a digital file the autobiographical script of their traumatic experience. After some days they returned to the therapist for the first EMDR session (T0). Before the session started in the presence of a trained psychologist (GN) all subjects filled in 3 self-administered neuropsychological checklists whose completion required about 30 minutes. They were then invited to walk into the therapy room where the EEG cap was positioned.

EEG recording was continuously performed while the patients were:

at rest with eyes open and closed;
listening to the script with eyes closed;
during a second period with eyes closed;
during EMDR therapy;
during a final period of rest.

**Figure 1:**
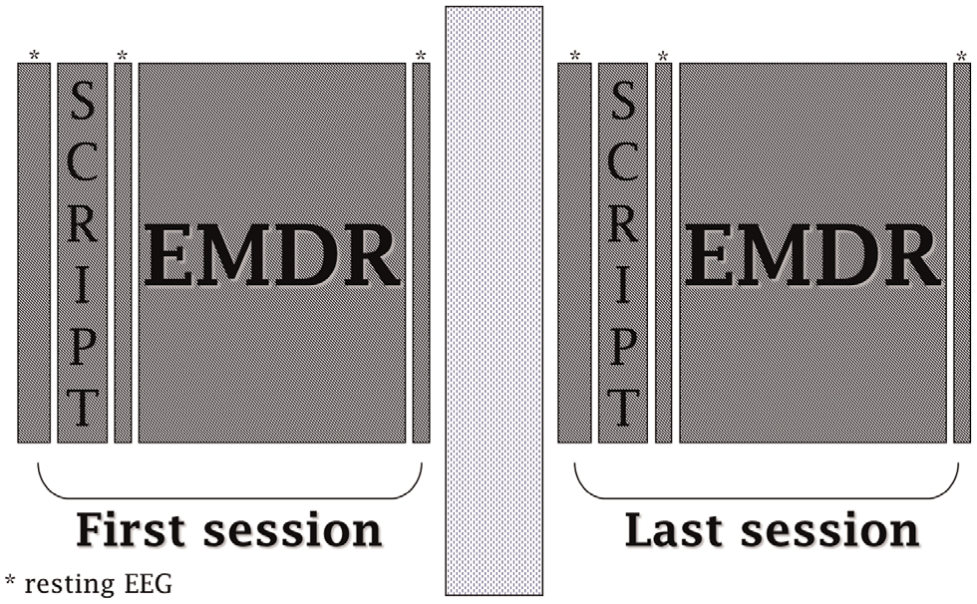
Study design.

The same protocol was repeated during the last EMDR session (T1), after the patient completely processed the trauma and reported no disturbance with Subjective Unit of Distress (SUD) = 0, Validity of Cognition (VOC) = 7 and clear Body Scan (Figure 1). A clinical follow-up of two years was then performed with all patients.

Control subjects underwent the same therapeutic protocol and neuropsychological assessments as patients but the EEG recording of the script and of the whole EMDR session were performed only on one occasion, right after the initial neuropsychological assessment (T0).

### EMDR Procedure

At the beginning of EMDR sessions patients and controls were asked to focus on the primary elements of the traumatic memories while at the same time following a "dual stimulation" using bilateral ocular stimulation (BS) lasting usually 30 seconds and entailing about 30 complete horizontal left-right-left eye movements each. Progressive changes after BS sets reflect reprocessing of the memory, until patients are able to engage in the recollection of the event with no disturbing emotions and with positive and constructive perspectives about themselves, showing desensitization and experience adaptive resolution. Once the memory of the traumatic event has been reprocessed, the EMDR protocol is applied to recent triggers and to future anxiety provoking or avoidance situations. Treatment will be completed as soon as past, present and future trauma-related issues are addressed. Treatment completion is usually associated with post-traumatic symptoms reduction.

### Treatment’s Eight Phases

Phase one is devoted to history taking. During phase one the clinician assesses symptoms and makes a diagnosis. At this time the patients’ readiness for EMDR is evaluated and a treatment plan is carried out. Patients along with the therapist identify possible EMDR processing targets. During phase two the therapist ensures that patients are provided with adequate resources for handling emotional distress and good coping skills. Patients are then prepared to start processing traumatic material, by explaining the method and showing BS, while focusing on a positive memory (safe place exercise). From phase 3 through 6, a target is identified and processed using the EMDR protocol. These phases involve patients identification of the most vivid visual image related to the memory (if available), a positive and a negative belief about the self, related emotions and body sensations. In the desensitization phase (Phase 4) patients are instructed to focus on the image, negative belief and body sensations while simultaneously moving their eyes back and forth following the therapist's fingers as they move across their field of vision for 20–30 seconds or more. This is repeated numerous times throughout the session. When patients report no distress related to the targeted memory, the clinician asks them to think of their preferred positive belief and to focus on the incident, while simultaneously engaging in further sets of eye movements. After several sets, patients generally report increased confidence in this positive belief. The therapist checks the patients’ body sensations. If there are negative sensations, these are processed as above. If there are positive sensations, they are further enhanced. In the closure phase (phase 7), the therapist instructs the patients how they should focus their attention after the session and ask them to keep a weekly log and to write down any related material that may arise. The therapist finally reminds the patients of the self-calming activities that were mastered in phase two. The next session begins with phase eight, i.e. reviewing the work done and checking whether results are maintained from the previous session.

### Self-administered Checklists

IES [17] is a 15-item checklist used to measure the psychological response to stressful or traumatic life events during the previous week. It specifically tackles the areas of intrusion (7-items subscale) and avoidance (8-items subscale) as key features of dysfunctional psychological adaptation following traumas. Scores above 26 are regarded as clinically significant.

BDI [18] is a 21-item self-report measure containing items related to the cognitive, affective as well as somatic symptoms of depression. Items are rated between 0, not at all, and 3, severely, in terms of how much they have bothered patients in the previous week. Scores above 18 indicate moderate to severe depressive symptoms.

SCL-90 R [19] is a 90-item self report symptom inventory used as a measure of psychological problems assessing the frequency of a broad range of symptoms of psychopathology. Patients rate the 90 items using a 5-point scale (1 = no problem to 5 = very severe) to measure the extent to which they have experienced the shortlisted symptoms over the last 7 days. The SCL-90-R has also 3 global indexes: the Global Severity Index (GSI) measures the extent or depth of the individual’s psychiatric disturbance; the Positive Symptom Total (PST) counts the total number of questions rated above 1 point; and the Positive Symptom Distress Index (PSDI) represents the intensity of symptoms.

Paired and un-paired t-tests were performed to compare the scores of IES, BDI, SCL-90-R between patients pre- and post-EMDR treatment and patients to controls, respectively.

### EEG Procedure

#### EEG acquisition

Thirty-seven-channel EEG was recorded using a pre-cabled electrode cap (Bionen, Florence, Italy). A horizontal electro-oculographic (H-EOG) channel, recorded from two electrodes at the outer canthus of each eye, was used to monitor eye movements of BS. The electrodes cup montage required approximately 20 minutes and was well tolerated by subjects. Electrode impedances were kept less than 10 KΩ. The signal was amplified by 40-channel EEG device (Galileo MIZAR-sirius, EBNeuro, Florence, Italy) and acquired with GalNT software. Data were collected with a sampling rate of 256 Hz and with hardware EEG filters of High-Pass at 0.099 Hz and Low-Pass at 0.45 SR (0.45×256 Hz = 115.2 Hz).

#### Preprocessing

Data were exported to EDF using NPX Lab 2010 (www.brainterface.com). In both patients and controls while the script recordings were fully exported, in the EMDR arm we segmented and exported only the BS periods (eliminating, arbitrarily, the first four and the last two eye movements), creating files of 180 seconds each with concatenated/merged periods of BS.

Data were analyzed in the EEGLAB environment (http://www.sccn.ucsd.edu/eeglab/index.html) a collection of scripts running under Matlab 7.7.0 R2010a (Mathworks Inc., Natick, MA). After visual inspection and manual elimination of paroxysmal artifact periods, artifact non-cerebral source activities (eye blinks and movements, cardiac and muscle/electromyographic activity) were identified and rejected using a semiautomatic procedure based on Independent Component Analysis [20].

#### Electrical Source Imaging (ESI)

To compute the intracerebral electrical sources underlying EEG activity recorded at the scalp we used the exact low resolution brain electromagnetic tomography (eLORETA) software (http://www.uzh.ch/keyinst/loreta.htm). Computations were made in a realistic head model [21], using the Montreal Neurological Institute (MNI; Montreal, Quebec, Canada) MNI152 template, with the three-dimensional solution space restricted to cortical gray matter and hippocampi, as determined by the probabilistic Talairach atlas [22]. The intracerebral volume (eLORETA inverse solution space) is partitioned in 6239 voxels at 5 mm spatial resolution (i.e., cubic elements of 5×5×5 mm). Anatomical labels as Brodmann areas are also reported using MNI space, with correction to Talairach space [23]. We calculated eLORETA images corresponding to the estimated neuronal generators of brain activity within each band [24]. The ranges of the frequency bands were as follows: delta (δ), 1.5–4 Hz; theta (θ), 4–8 Hz; alpha (α), 8–12 Hz; beta 1 (β1), 12–20 Hz; beta 2 (β2), 20–30 Hz; gamma (γ), 30–45 Hz.

#### Statistics

eLORETA software package was used to perform ESI statistical analyses. The methodology used was non-parametric randomization statistics (Statistical non-Parametric Mapping, SnPM) [25]. A second level of non-parametric analysis, the exceedence proportion tests evaluated the significance of activity based on its spatial extent, obtaining clusters of supra-threshold voxels.

Between-group comparisons of the eLORETA current density distribution were performed using a statistical analysis based on voxel-by-voxel log of F ratio test with 5000 randomizations. The results corresponded, for each band, to maps of log-F-ratio statistics for each voxel, for corrected p<0.05. Significant activations at the exceedence proportion tests with a p value <0.01, F value over 2 z-score and a minimum cluster of voxels major than 27 (an intracerebral volume cube with an edge of 15 mm) within a hemisphere for single Broadmann Area (BA) were accepted.

#### Electrical source functional connectivity

Functional connectivity analysis was performed by the “whole-brain Brodmann areas (BAs)” approach, using the anatomical definitions of 84 BAs provided by eLORETA software package and based on the Talairach Daemon (http://www.talairach.org/). Pairs of BAs were analyzed using the values of single voxels with the highest F-ratio value at the centroid of each BA. To test interregional functional correlations between any pair of BAs lagged phase synchronization (LPS), index of physiological lagged connectivity and decomposing connectivity into instantaneous and lagged components, was used [26], [27]. It defines the phase synchronization between two signals in the frequency domain based on normalized Fourier transforms after partialling out the instantaneous, zero-lag contribution resulting from non-physiological effects or intrinsic artifacts. Hence, this measure is thought to contain physiological connectivity information only.

Since no significant correlation was found for any of the 42 pairs, we selected 21 BAs (9, 10, 11, 17, 18, 19, 20, 23, 24, 25, 28, 30, 31, 32, 33, 34, 35, 36, 37, 46, 47), bilaterally, and repeated the analyses without obtaining any significant result. Finally we chose an *a priori* approach further reducing the number of tested regions to the clusters in which for each group comparison significant differences were found. The latter analyses were performed averaging for each of the six EEG bands, the LPS values in all voxels within a sphere of 15 mm of radius around the one with maximal intensity.

Statistical comparisons were carried out using non-parametric randomization techniques with correction for multiple comparisons.

#### Correlation with neuropsychological scores

To evaluate the association between connectivity measures and neuropsychological variables, the LPS values of the pairs of clusters found to have significantly changed were correlated to the scores of IES-total, BDI total and SCL-90R-PSDI, yielding r, r^2^ and p values corrected for multiple comparisons.

## Results

### Self-administered Checklists

In all patients after 3 to 8 EMDR sessions (mean 5) symptoms related to the traumatic event disappeared and SUD and VOC scores reached the normal values of 0 and 7 respectively. All patients were still symptoms-free after 2 years of follow up. Scores of IES, BDI and SCL-90-R were significantly different between patients and controls at T0 (Table 1) and decreased significantly in patients at T1 (Table 2).

**Table 1:** Pre EMDR treatment: mean (SD) and statistically significant differences in IES, BDI and SCL-90-R scores in patients *vs* controls.

	Patients (N = 10)	Controls (N = 10)	T	p
IES/pre/TOTAL	40.8 (15.9)	2 (3.1)	7.543	0.000
IES/pre/intrusion	21.1 (9.8)	1 (2.2)	6.297	0.000
IES/pre/avoidance	19.7 (7.7)	1 (1.3)	7.536	0.000
BDI/pre/TOTAL	23.9 (10.1)	1.6 (2.2)	6.795	0.000
BDI/pre/cognitive	15.7 (8.1)	0.70 (1.3)	5.799	0.000
BDI/pre/somatic	8.2 (3.3)	0.90 (1.3)	6.416	0.000
SCL-90-R/pre/PST	59.6 (20.2)	6.2 (6.35)	7.956	0.000
SCL-90-R/pre/PSDI	2.11 (.53)	0.82 (.61)	4.989	0.000
SCL-90-R/pre/GSI	1.49 (.65)	0.80 (.80)	6.293	0.000

**Table 2:** Pre *vs* post EMDR treatment: mean (SD) and statistically significant differences in IES, BDI and SCL-90-R scores in patients.

	Patients (N = 10)	T	p
IES/pre/TOTAL *vs* IES/post/TOTAL	40.8 (15.9) *vs* 12.8 (12)	6.386	0.000
IES/pre/intrusion *vs* IES/post/intrusion	21.1 (9.8) *vs* 6.6 (6.6)	5.7	0.000
IES/pre/avoidance *vs* IES/post/avoidance	19.7 (7.7) *vs* 6.3 (5.9)	5.448	0.000
BDI/pre/TOTAL *vs* BDI/post/TOTAL	23.9 (10.1) *vs* 9.5 (9.5)	4.003	0.003
BDI/pre/cognitive *vs* BDI/post/cognitive	15.7 (8.1) *vs* 6.7 (7.1)	3.085	0.013
BDI/pre/somatic *vs* BDI/post/somatic	8.2 (3.3) *vs* 2.8 (2.6)	4.92	0.001
SCL/pre/PST *vs* SCL/post/PST	59.6 (20.2) *vs* 37.7 (19.7)	4.948	0.001
SCL/pre/PSDI *vs* SCL/post/PSDI	2.11 (.53) vs 1.41 (.46)	3.625	0.006
SCL/pre/GSI *vs* SCL/post/GSI	1.49 (.65) vs 0.66 (.52)	4.131	0.003

### EEG

#### Patients vs. controls

**Figure 2:**
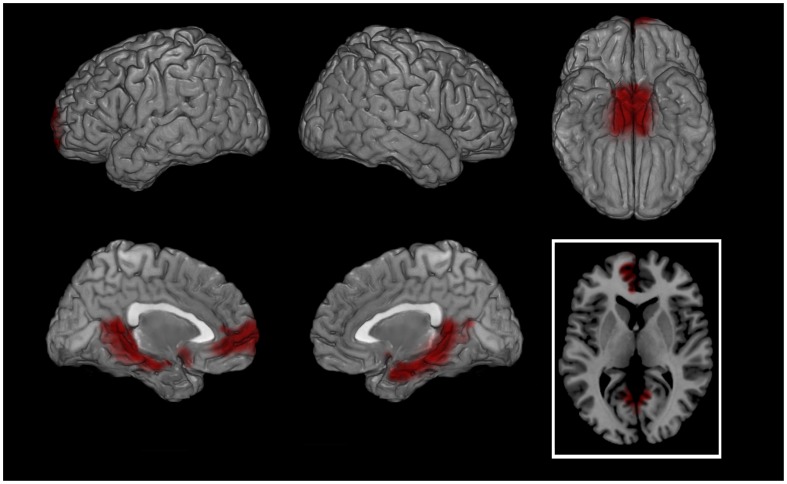
SCRIPT: PATIENTS - CONTROLS (theta band). Cortical representation of the cluster of voxels in which the EEG signal showed significant differences between groups. Activation increases exceeding a p value < 0.01 and an F value over 2 z-score are depicted by red color scale. Top row left: lateral view of left hemisphere; Top row middle: lateral view of right hemisphere; Top row right: view from below; Bottom row left: medial view of left hemisphere; Bottom row middle: medial view of right hemisphere; Bottom row right: transversal section at prefrontal cortex level level (z = 5). Regional details are presented in Table 3.

**Figure 3:**
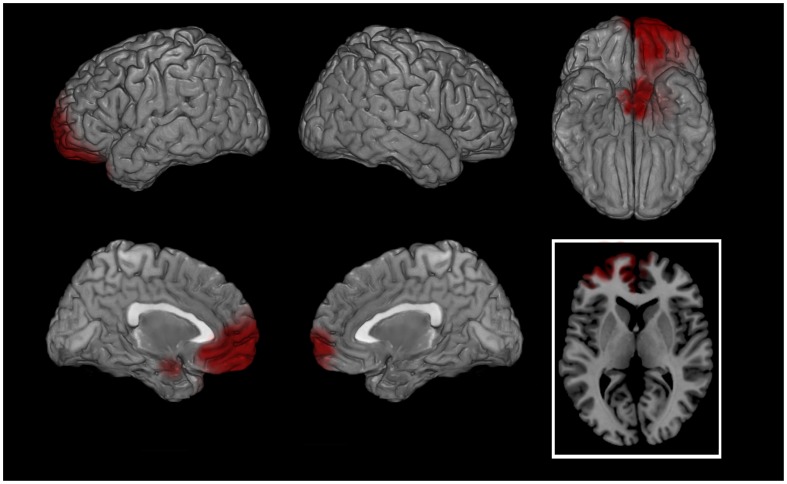
EMDR BS: PATIENTS - CONTROLS (gamma band). Cortical representation of the cluster of voxels in which the EEG signal showed significant differences between groups. Activation increases exceeding a p value <0.01 and an F value over 2 z-score are depicted by red color scale. Top row left: lateral view of left hemisphere; Top row middle: lateral view of right hemisphere; Top row right: view from below; Bottom row left: medial view of left hemisphere; Bottom row middle: medial view of right hemisphere; Bottom row right: transversal section at prefrontal cortex level level (z = 5). Regional details are presented in Table 3.

During the script a significantly higher cortical activation was found in patients’ bilateral orbito-frontal cortex (OFC, BAs 11–47) and anterior cingulate cortex (ACC, BAs 24-25-32-33) for almost all frequencies between 1.5 and 20 Hz (Table 3). Significantly higher bilateral activation was also found for delta and theta bands bilaterally in parahippocampal gyri (PHG, BAs 28-34-35-36) and for theta band in bilateral posterior cingulate cortex (PCC, BAs 23-30-31) (Table 3; Figure 2). During BS patients showed a higher cortical activation in left OFC, rostral prefrontal cortex (rPFC, BA 10) and ACC for most of the bands (Figure 3). Significantly higher activations in patients were also found for some bands in PHG and PCC (Table 3).

**Table 3:** Regions in which significant differences were found between different conditions and groups.

	BA	δ		θ		α		β1		β2		γ	
		left	right	left	right	left	right	left	right	left	right	left	right
**SCRIPT PATIENTS –** **CONTROLS**	**OFC**	76	35			65	29	70	27				
	**ACC**	40	38			47	36	44	35				
	**PHG**	35	56	48	42			39					
	**PCC**			34	58								
**BS PATIENTS - CONTROLS**	**OFC**	59				77				113		155	
	**rPFC**	72		48		72		46		91		97	
	**ACC**	34				30				39		44	
	**PHG**	33	37		41			36				32	
	**PCC**				29		34	32	56				
**SCRIPT PATIENTS T1 –** **PATIENTS T0**	**FG**		48		104		50		134				
	**VC**	212	46		41								
**BS PATIENTS T1 –** **PATIENTS T0**	**FG**	108				81		88		127		115	
**BS PATIENTS T0 ** ***–*** **PATIENTS T1**	**rPFC**			37		40	27	44					
	**VC**				33		42		43				

BS = bilateral ocular stimulation during EMDR therapy; δ = delta, 1.5–4 Hz; θ = theta, 4–8 Hz; α = alpha, 8–12 Hz; β = beta 1, 12–20 Hz; β2 = beta 2, 20–30 Hz; γ = gamma, 30–45 Hz; OFC = orbito-frontal cortex (BAs 11–47); ACC = anterior cingulate cortex (BAs 24-25-32-33); PHG = parahippocampal gyrus (BAs 28-34-35-36); PCC = posterior cingulated cortex (BAs 23-30-31; FG = fusiform gyrus (BAs 20–37); VC = visual cortex (BAs 17-18-19); rPFC = rostral prefrontal cortex (BA 10). For significant comparisons the number of voxels in each cluster is reported for each band and each hemisphere.

#### Patients at T1 vs. patients at T0

**Figure 4:**
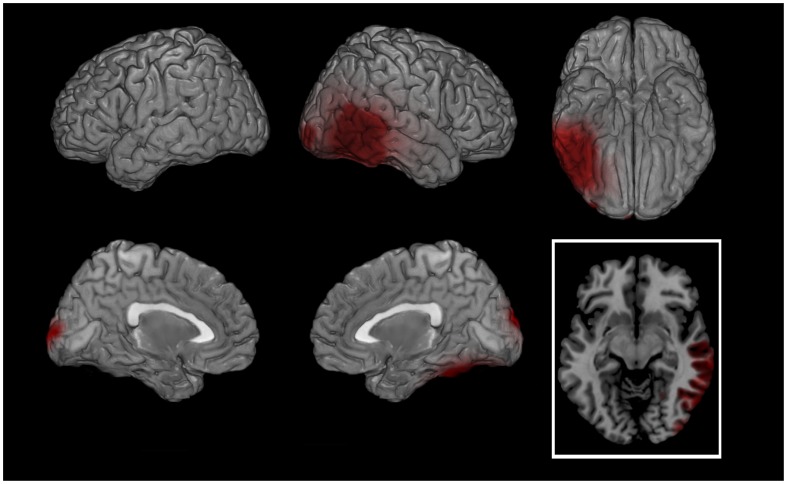
SCRIPT: PATIENTS T1 - PATIENTS T0 (theta band). Cortical representation of the cluster of voxels in which the EEG signal showed significant differences between conditions. Activation increases exceeding a p value <0.01 and an F value over 2 z-score are depicted by red color scale. Top row left: lateral view of left hemisphere; Top row middle: lateral view of right hemisphere; Top row right: view from below; Bottom row left: medial view of left hemisphere; Bottom row middle: medial view of right hemisphere; Bottom row right: transversal section at temporal cortex level (z = −10). Regional details are presented in Table 3.

**Figure 5:**
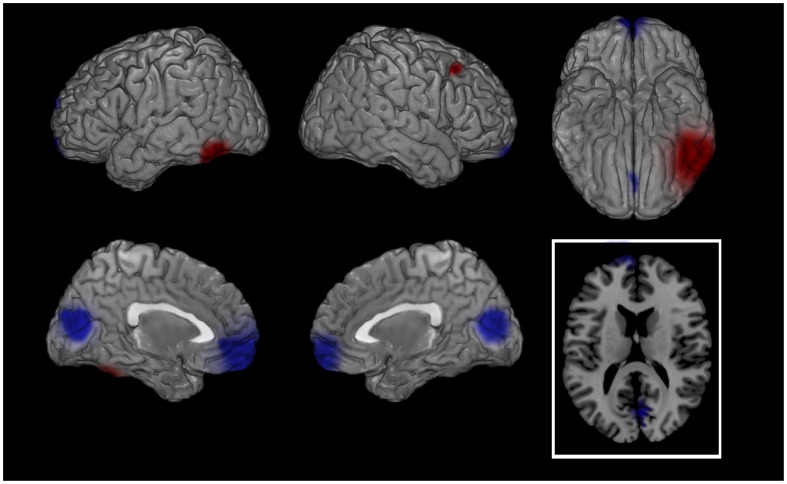
EMDR BS: PATIENTS T1 - PATIENTS T0 (alpha band). Cortical representation of the cluster of voxels in which the EEG signal showed significant differences between conditions. Activation increases exceeding a p value <0.01 and an F value over 2 z-score are depicted by red color scale; activation decreases are depicted by blue color scale. Top row left: lateral view of left hemisphere; Top row middle: lateral view of right hemisphere; Top row right: view from below; Bottom row left: medial view of left hemisphere; Bottom row middle: medial view of right hemisphere; Bottom row right: transversal section at primary visual cortex level (z = 15). Regional details are presented in Table 3.

During the script listening there was a significantly higher cortical activation in patients at T1 in right fusiform gyrus (FG, BAs 20-37) for bands up to 20Hz. A higher activation was also recorded at T1 in visual cortex for delta and theta bands (Figure 4). During BS a significantly higher left FG activation was found at T1 for all but theta bands (Table 3). In this comparison a significantly higher activation was found at T0 as compared to T1 in rPFC, mainly on the left, and in right visual cortex (Figure 5) in the frequencies between 3 and 20Hz.

#### Functional connectivity

At connectivity analysis a significantly decreased pair-wise interaction as expressed by LPS between left VC and right FG was found in patients at T1 as compared to T0 during the script listening in the theta band.

Significantly decreased functional connectivity was also found in patients in the gamma band during bilateral ocular stimulation in comparison with controls in two pair-wise interactions: left PFC and left PCC; left ACC and left PCC.

#### Correlation with neuropsychological scores

The scores of the neuropsychological tests in patients were not only consistent with symptom remission as assessed clinically and by SUD and VOC but they also correlated significantly with LPS in the pair-wise interactions found to be significantly changed. The pathological pre-EMDR and normalized post-EMDR scores of IES total, BDI total and SCL-90-R PSDI, taken as a *continuum*, showed a significantly positive correlation in theta band with the LPS values of the pair-wise interaction between left VC and left FG during script listening in patients at T1 *vs* T0. Negative correlations in the gamma band between LPS values and the same neuropsychological tests scores were found for the other two pair-wise interactions found to be significantly decreased during BS in patients as compared to controls: left PFC *vs* left PCC; and left ACC *vs* left PCC (Table 4).

**Table 4:** Correlations between Lagged Phase Synchronization (LPS) indexes and psychometric variables.

	Changed pair-wise interactions *vs* neuropsychological tests	r	r^2^	p
**SCRIPT T1 ** ***vs*** ** T0 (theta band)**	left VC – right FG *vs* IES	0.531	0.282	0.016
	left VC – right FG *vs* BDI	0.505	0.255	0.023
	left VC – right FG *vs* PSDI	0.484	0.235	0.030
**EMDR patients ** ***vs*** ** controls (gamma band)**	left PFC – left PCC *vs* IES	−0.666	0.443	0.001
	left PFC – left PCC *vs* BDI	−0.594	0.353	0.006
	left PFC – left PCC *vs* PSDI	−0.550	0.302	0.012
	left ACC – left PCC *vs* IES	−0.644	0.415	0.002
	left ACC – left PCC *vs* BDI	−0.567	0.322	0.009
	left ACC – left PCC *vs* PSDI	−0.493	0.243	0.027

## Discussion

The first relevant result of the study was the ability to perform an on-line monitoring of the cortical firing occurring during EMDR therapy by means of the EEG, more specifically during bilateral ocular stimulation. For the first time, maximal brain activations associated with the therapeutic actions envisaged by the EMDR protocol could be outlined and represented on the cortical surface. To the best of our knowledge this is also the first time psychotherapy is monitored and dynamically represented by functional imaging throughout its entire duration. The logistic and technical effectiveness of such complicated methodology carried out by psychotherapists, psychologists, psychiatrists and EEG technicians, all at the same time, provided the opportunity of performing the experiments in a totally patient-friendly environment, i.e. in a comfortable private practice therapy room, avoiding possible biases resulting from physical and psychological discomfort for the patient due to an unfriendly examination environment [28].

Following successful EMDR therapy, the main neurobiological finding of the study was the shift of the maximal cortical firing, during both autobiographic script listening and BS, from prefrontal and limbic regions at T0 to fusiform and visual cortex at T1 (Figure 4 and 5, respectively). Also when compared to asymptomatic normal subjects the reliving of the major traumatic event caused in patients a significantly higher bilateral limbic firing during the script (Table 3; Figure 2) and a more leftward oriented limbic activation during BS (Figure 3). The latter finding might be related during BS to the guided attempt to encode unelaborated emotional material, activating preferentially left rPFC [29].

The significantly higher activation found in patients during the BS at T0 compared to T1 in rPFC (Figure 5) confirms the leftward differences found during the same phase in patients as compared to controls (Figure 3). Prefrontal activation is associated with evaluation of self-generated material [30] being anterior cingulate cortex the point of integration of emotional information involved in the regulation of affect [31] as well as a key substrate of conscious emotional experience monitoring information with affective consequences. Rostral PFC as part of the limbic system is thought to be involved in processes concerning the emotional value of incoming information and to be critically implicated in functions altered in psychic trauma response. Its activation upon emotional induction is considered to represent the neurobiological correlate of the affective valence of the stimulus [32]. Moreover, episodic memory retrieval is known to activate PFC [33], and a close relationship between autobiographical/episodic memory, the self and the involvement of PFC was described [34]. PFC has also been found to be activated while suppressing unwanted memories [35] and was found by near infrared spectroscopy to be activated during trauma recall before EMDR therapy [36]. All these functions may be exaggerated in patients before EMDR therapy in which the self-referential emotional contents cause an activation in rPFC larger than in normal individuals or in the patients after having processed the traumatic event.

One relevant neurobiological effect of EMDR in patients was represented by the differences found between the cortical activation at T0 as compared to T1 during script listening (Figure 4). In this comparison we found at T1 a significant increase of the EEG signal in right FG as well as in right visual cortex (VC). These changes suggest a better cognitive and sensorial (visual) processing of the traumatic event during the autobiographic reliving after successful EMDR therapy with a preferential activation moving from the emotional fronto-limbic cortex (at T0) towards the associative temporo-occipital cortex (at T1). Once the memory retention of the traumatic event can move from an implicit subcortical to an explicit status different cortical regions participate in processing the experience. On the other hand FG is implicated in the explicit representation of faces, words and abstract thoughts [37] and its prevalent activation after successful EMDR therapy might be associated with an elaboration at higher cognitive level of the images related to the event.

As found in the script analysis, FG showed a higher activation also during BS at T1. Interestingly, in our patients these comparisons showed different outcomes with a clear lateralization towards the left hemisphere during BS (Figure 5) and on the right side during the script listening (Figure 4). According to the emotional asymmetry theory the right hemisphere is dominant over the left for emotional expressions and perception. Furthermore, both hemispheres function as somewhat of a functional unit and an increased activation in one of them will result in an inhibition in the contralateral one. The prominent activation found during BS at T1 in association areas in left hemisphere might then correspond to a cognitive processing of traumatic memories reaching the explicit state after successful EMDR therapy associated to a significant restraint of negative emotional experiences. The left hemisphere has also an important role in explicating emotions and left FG was also found to be activated during tasks implying episodic memory and memory retrieval associated with attentional control [37].

The differential neuronal firing at T0 in patients as compared to control subjects (Figure 2 and 3) not only highlighted the emotional component of the trauma retrieval when patients were still symptomatic but also ruled out the possibility that these regions were activated merely due to the reliving of the index event. Furthermore in both script and BS patients activated more than controls PHG and ACC, being the latter the neural link of the former with PFC. The primary difference between patients and controls was not due to the nature of traumas but to the lack of symptoms in the latter. Physical and/or psychological traumas cause anxiety states based not only on severity but also on personality, on life-time trauma load and probably on genetic factors associated to each individual person.

After progressively reducing the number of investigated regions out of the clusters resulting significantly different in the group comparisons, interregional connectivity changes reported while reliving of the traumatic event, representing the variations in brain activity networking upon different conditions, were found in three cluster pairs. The loss of functional connectivity between left VC and FG found in patients at T1 as compared to T0 during the script listening was associated with the disappearance of symptoms and speaks in favor of disconnection of a pathological visual network after successful EMDR therapy. At this stage, as an effect of successful trauma elaboration, the visual images of the event are processed and stored in primary and associative visual cortex and likely decoupled from the emotional memory of faces and bodies linked to the event, typically processed by FG. Moreover, affectively valenced stimuli were shown to prompt event-related synchronization in posterior brain regions in the theta frequency band [38]. Such synchronization might have disappeared once the images of the traumatic event lost their emotional meaning.

The findings of decreased pair-wise interactions between PFC, ACC and PCC found in patients as compared to controls during BS show that the functional connectivity during trauma relieving and involving three important frontal regions was not present in patients. This underscores the pathological nature of the changes occurring in post-traumatic conditions in the limbic system and the central role of the latter in properly processing negative autobiographical events. Event-related activity in gamma band was observed in healthy volunteers in ACC and left PFC upon exposition to emotional stimuli [39], suggesting that gamma activity in PFC may be modulated by emotional processing in ACC. Furthermore gamma band seems to reflect short distance synchrony [40].

The relative low number of significant pairs-wise interactions found in the present study is probably due to the limited number of investigated subjects (and hence to lesser statistical power). The constraint to restrict the amount of regions from the 84 eLORETA default ones to the clusters resulting significant in group comparisons was due to multiple comparisons corrections, cutting down dramatically on the significance of each analysis. When more patients and controls are available a dedicated study aiming specifically at investigating functional connectivity will be possible.

Comparing our findings to previous studies investigating psychological traumas [41], significantly higher activations in OFC and rPFC in patients during script were found by some [13], [42]–[44], but not by other authors [45], [46]. SPECT studies have also investigated the effect of psychotherapies and pharmacologic treatment on CBF reporting both increases and decreases distributed throughout the whole cortex [11], [13], [47], [48]. Functional studies in psychological trauma employ different methodologies varying from analyzing resting brain activity to the implementation of stimuli and active tasks, including scripts. Moreover, patients with broad trauma spectra and types are recruited resulting in different brain activation patterns. Due to this heterogeneity, comparing data across studies is difficult especially when different methodologies are implemented as in the case of this pioneering EEG study.

All bands showed significantly different changes across the four performed comparisons especially at frequencies between 1.5 and 20 Hz (Table 3). The significant differences in theta frequency were mostly found during the autobiographical script analyses (Table 3; Figure 2 and 4). Hippocampal theta rhythm is implicated in episodic memory [49] and memory formation and retrieval [50] and has been found to correlate with neuronal firing in frontal cortex [51], [52]. Furthermore increased theta activity localized in hippocampus was found in one of the few studies investigating EEG in PTSD [53] supporting the evidence of its role in modulating emotional memories.

Another interesting finding of the study is the significant difference in gamma band between patients and controls during BS (Table 3, Figure 3). Such difference, localized in frontal cortex and PHG during the effort to encode unprocessed emotional material, is consistent with previous studies on gamma synchronicity in which neuronal firing in frontal cortex was associated with behaviorally relevant sensory information and highly alert brain states [54]. On the other hand, attention was associated with reduced alpha rhythms [55] and the latter negatively correlated with behavioral performances in non-human primates [56] having also an active role in inhibiting unattended information in attentional tasks [57]. The finding of a significantly lower alpha band activity in frontal cortex at T1 (Figure 5) supports the hypothesis that after trauma processing with EMDR the traumatic event *per se* will be under control through a more attentive cognitive-associative modes. In this respect also the prominent beta band activation in limbic regions (OFC, rPFC, ACC, PHG and PCC) can be interpreted as increased selective attention and perception of the index trauma in patients as compared to controls [58].

Delta waves were significantly higher in patients as compared to controls and in patients at T1 as compared to T0 in all regions involved in the EMDR related changes (Table 3). Their increase in association with BS can tentatively be ascribed to the oscillation caused by such slow-wave-sleep-like stimulus [59] and their increase in frontal cortex of patients as compared to controls might be related to thought processes under unusual conditions [60].

A recent theory postulates that traumatic memories are retained in amygdalar synapses due to powerful electric signals overpotentiating alpha-amino-3-hydroxy-5-methyl- 4-isoxazole (AMPA) receptors. During slow wave sleep (SWS) this would prevent their merging with the cognitive memory trace via anterior cingulate cortex (for review see [59]). Animal studies have demonstrated that a low-frequency tetanic stimulation using one to five pulses per second can cause in the synapses of the basolateral tract of the amygdale a depotentiation of AMPA receptors proportional to the stimulation frequency and extinguishing the traumatic memories [61].

Such stimulus is similar to the one administered during EMDR sessions (about 2 Hz) and the pathophysiological mechanism of the therapy might be related to the slowing of the depolarization rate of neurons in the limbic system elicited by BS. This in turn would result in the emotional memories pathologically confined in the amygdale moving to higher brain centers and being fully processed [59]. At macroscopic level, our findings (hyperactivation of parahippocampal gyrus and limbic cortices at T0 in both BS and script listening) seem to support such hypothesis even if in humans functional studies focused on neuronal firing, finer spatial identification and time resolution are needed to better investigate this fascinating issue.

According to the Adaptive Information Processing theory [62] when a traumatic event occurs, information processing may be incomplete, probably due to the fact that strong negative feelings or neurobiological reactions interfere with it. This prevents the forging of associative connections of memory with other networks and memory is dysfunctionally stored. During an EMDR session memory distressing components are linked to more adaptive information existing in the neural networks and therefore memory desensitization and reprocessing take place, thus contributing to symptom reduction and ultimately remission.

The assessment of severity and persistence of trauma related symptoms is of paramount importance. IES and BDI are commonly administered pre- and post-treatment as measures of outcome and this approach is particularly evident in studies on effectiveness of psychotherapy for traumatised patients [9], [63].

The dramatic decrease of IES from moderate impact to sub-cut-off scoring was such for both intrusion and avoidance subscales indicating the efficacy of EMDR sessions on both components. The same held true for BDI in which scores moved from moderate depression to sub threshold values between minimal and mild depression ranges for both cognitive and somatic components.

We found significant positive correlations between the functional connectivity changes (as expressed by lagged phase synchronization values) in patients at T1 as compared to T0 in VC and FG and the scores of neuropsychological tests during script listening and negative correlations of the same scores and some regions of the frontal and parietal limbic system in which reciprocal connectivity changed significantly (PFC, ACC and PCC) during BS when comparing patients and controls (see Table 4).

The different directions of the correlations are due to the fact that LPS represented in the former case a decreased connectivity between patients at T1 as compared to patients at T0 (lower LPS, lower neuropsychological scores) whereas in the latter case control subjects showed higher connectivity (higher LPS, lower neuropsychological scores).

Such correlations highlight the association between three important dimensions of the pathological and diagnostic processes (i.e. functional changes, neuropsychological assessment and clinical status) and confirm the neurobiological ground and effects of EMDR therapy. Statistical significance was achieved in the correlations of tests scores of IES total, BDI total and SCL-90R-PSDI with all the investigated pair-wise interactions confirming the role of the above neuropsychological tests in the diagnosis and clinical assessment of post-traumatic conditions. In the future more studies with a larger number of subjects are needed to highlight the correlations between such scores and other regions, but more importantly to identify the sites of neural representations and/or processing of the above constructs under post-traumatic conditions [64].

EMDR sessions, seemed also to spread positive effects on a general reduction of psychiatric symptoms associated with the posttraumatic condition, quite the rule in individuals who have experienced multiple and repeated traumas [65]. In this respect, the literature shows a compelling evidence of what Bremner [66] has described as “trauma spectrum psychiatric disorders” including mild to severe depression and anxiety disorder [67], [68]. Our findings seem to follow this vein since in patients all three scores of global index of distress of SCL-90-R were significantly changed after EMDR therapy. The striking decrease in depression as measured by the BDI and in the quantity and quality of symptoms as measured by the SCL-90-R has to be regarded as a further indication of EMDR treatment efficacy in tackling and ameliorating psychiatric disorders in the trauma spectrum.

One of the constraints of the study is the relatively small number of investigated subjects. However numerousness lies in the magnitude of the neuroimaging study in which the high costs and the complicated methodologies limit the amount of subjects to be studied. On the other hand recruitment of controls increased the robustness of the results adding a between-subjects analysis to the comparison of patients at T0 and T1. Finally, a systematic and exhaustive discussion of all differences found in each EEG band (Table 3) was beyond the scope of the present study and we have deliberately confined the discussion to some of the most relevant results.

### Conclusions

Our findings point to a highly significant activation shift following EMDR therapy from limbic regions with high emotional valence to cortical regions with higher cognitive and associative valence. This suggests a strong neurobiological rationale of EMDR, thus supporting its efficacy as an evidence- based treatment for trauma. On the other hand the pathophysiological changes occurring during EMDR psychotherapy were monitored on-line for the first time, confirming the validity of the proposed EEG methodology and encouraging further studies with a larger cohort of subjects.
